# Elevated ubiquitinated proteins in brain and blood of individuals with schizophrenia

**DOI:** 10.1038/s41598-019-38490-1

**Published:** 2019-02-19

**Authors:** Chad A. Bousman, Sandra Luza, Serafino G. Mancuso, Dali Kang, Carlos M. Opazo, Md. Shaki Mostaid, Vanessa Cropley, Patrick McGorry, Cynthia Shannon Weickert, Christos Pantelis, Ashley I. Bush, Ian P. Everall

**Affiliations:** 1 Melbourne Neuropsychiatry Centre, Department of Psychiatry, The University of Melbourne & Melbourne Health, Carlton South, VIC Australia; 2 The Cooperative Research Centre (CRC) for Mental Health, Victoria, Australia; 3 Melbourne Dementia Research Centre, Florey Institute of Neuroscience and Mental Health, The University of Melbourne, Parkville, VIC Australia; 4 Guangdong Food and Drug Vocational College, Guangzhou, China; 5 Orygen, The National Centre of Excellence in Youth Mental Health, Parkville, VIC, Australia; 6 Schizophrenia Research Laboratory, Neuroscience Research Australia, Baker Street, Sydney, Australia; 7 NorthWestern Mental Health, Melbourne, Victoria Australia; 8 School of Psychiatry, Faculty of Medicine, University of New South Wales, Sydney, Australia; 9 Centre for Neural Engineering, The University of Melbourne, Carlton, VIC Australia; 10 Institute of Psychiatry, Psychology and Neuroscience, King’s College London, London, United Kingdom; 11 Departments of Medical Genetics, Psychiatry, Physiology & Pharmacology, University of Calgary, Calgary, AB Canada; 12 Alberta Children’s Hospital Research Institute, University of Calgary, Calgary, AB Canada; 13 Hotchkiss Brain Institute, Cumming School of Medicine, University of Calgary, Calgary, AB Canada

## Abstract

Dysregulation of the ubiquitin proteasome system (UPS) has been linked to schizophrenia but it is not clear if this dysregulation is detectable in both brain and blood. We examined free mono-ubiquitin, ubiquitinated proteins, catalytic ubiquitination, and proteasome activities in frozen postmortem OFC tissue from 76 (38 schizophrenia, 38 control) matched individuals, as well as erythrocytes from 181 living participants, who comprised 30 individuals with recent onset schizophrenia (mean illness duration = 1 year), 63 individuals with ‘treatment-resistant’ schizophrenia (mean illness duration = 17 years), and 88 age-matched participants without major psychiatric illness. Ubiquitinated protein levels were elevated in postmortem OFC in schizophrenia compared to controls (p = <0.001, AUC = 74.2%). Similarly, individuals with ‘treatment-resistant’ schizophrenia had higher levels of ubiquitinated proteins in erythrocytes compared to those with recent onset schizophrenia (p < 0.001, AUC = 65.5%) and controls (p < 0.001, AUC = 69.4%). The results could not be better explained by changes in proteasome activity, demographic, medication, or tissue factors. Our results suggest that ubiquitinated protein formation may be abnormal in both the brain and erythrocytes of those with schizophrenia, particularly in the later stages or specific sub-groups of the illness. A derangement in protein ubiquitination may be linked to pathogenesis or neurotoxicity in schizophrenia, and its manifestation in the blood may have prognostic utility.

## Introduction

Protein homeostasis involves the regulation of protein formation (synthesis, folding, oligomerization), protein degradation, and peptide recycling (turnover)^1^. Protein turnover is controlled through two major pathways: the autophagosome-lysosomal system and the ubiquitin proteasome system (UPS)^2^, the latter tasked with identifying misfolded and foreign proteins.

The UPS comprises >1,500 proteins that play major roles in the proper function of a variety of basic cellular processes (e.g. neurotransmitter synthesis and receptor recycling, cytokine production and activation) that are perturbed in schizophrenia, and so may harbour potential pharmacological targets^3–5^. Empirically, the UPS has been linked to schizophrenia in genome-wide association^6^, microarray^7–13^, and protein^14–17^ studies in either blood or brain. Collectively, these studies have implicated a down-regulation of all components of the UPS in the blood or brain of individuals with schizophrenia but it is not clear which component(s) within the UPS, if any, are dysregulated in both blood and brain. Herein, we report the results of a study that measured free mono-ubiquitin, ubiquitinated proteins, catalytic ubiquitination, and proteasome activities in erythrocytes and postmortem orbitofrontal cortex (OFC) from individuals with schizophrenia and controls. We chose to examine erythrocytes because in their maturation from reticulocytes they lose all organelles including autophagosomes^18^ and so their only mechanism for protein degradation is via the UPS. Our focus on the OFC stemmed from our previous longitudinal work that showed significant reductions in OFC volume among individuals who developed psychosis^19^ as well as our post-mortem OFC studies suggesting marked interneuron pathology and neuroinflammation in schizophrenia^20–22^.

We hypothesized that the UPS would be dysregulated in both the OFC and erythrocytes of those with schizophrenia. In addition, we explored whether UPS markers in erythrocytes differed in those with recent-onset and treatment-resistant schizophrenia as this could provide a preliminary indication of whether UPS dysregulation best represents a marker of illness stage.

## Methods

### Participants

Frozen postmortem orbitofrontal cortex (OFC) tissue from 76 (38 schizophrenia, 38 control) individuals was obtained from the New South Wales Brain Tissue Resource Center (Sydney, Australia). In addition, we collected erythrocytes from 181 living participants, consisting of: (i) 63 individuals with treatment-resistant schizophrenia treated with clozapine, as these individuals did not respond to two or more previous trials of antipsychotics, had poor functioning, and persistent symptoms, they were considered ‘treatment-resistant’ (mean illness duration = 17 years) aligned with recent criteria^23^; (ii) 30 individuals with recent onset schizophrenia (mean illness duration = 1 year); and (iii) 88 healthy control participants. For details on tissue collection and processing see supplementary material.

All living participants were recruited from multiple clinical services and the community in Melbourne, Australia. Ascertainment and exclusions for the postmortem cohort have been published elsewhere^24^ and are described briefly in the supplementary material. Likewise, recruitment, inclusion, and exclusion details for the recent onset and ‘treatment-resistant’ schizophrenia cohorts have been recently described^25^ and are presented in detail within the supplementary material. Characteristics of each cohort are shown in Table 1.

**Table 1:** Clinical and postmortem brain cohort characteristics.

Characteristic	Clinical Cohorts							Postmortem brain cohort		
	1. Control	2. Recent onset	3. TRS	Omnibus	1 vs 2	1 vs 3	2 vs 3	Control	Schizophrenia	Omnibus
	n = 88	n = 30	n = 63	p	p	p	p	n = 38	n = 38	p
Age, mean (sd) years	35 (12)	21 (2)	40 (10)	**<0**.**001**	**<0**.**001**	**<0**.**001**	**<0**.**001**	52 (15)	52 (14)	0.824
Sex, % (n) female	35 (31)	24 (7)	22 (14)	0.207	—	—	—	26 (10)	34 (13)	0.618
Ethnicity, % (n) Caucasian	83 (73)	77 (23)	87 (53)	0.084	—	—	—	97 (37)	97 (37)	1
Current Smoker, % (n)	28 (19)	26 (18)	46 (32)	**<0**.**001**	0.880	**0**.**016**	0.061	24 (9)	61 (23)	**0**.**002**
Current alcohol user, % (n)	93 (82)	67 (20)	92 (58)	**<0**.**001**	**0**.**001**	0.516	**0**.**003**	47 (18)	53 (20)	0.646
Current cannabis user, % (n)	13 (11)	41 (12)	19 (12)	**0**.**005**	**<0**.**001**	0.246	**0**.**024**	—	—	
Age of onset, mean (sd) years	—	19 (2)	22 (6)	**0**.**001**	—	—	—	—	24 (7)	—
Duration of illness, mean (sd) years	—	1 (1)	17 (8)	**<0**.**001**	—	—	—	—	28 (14)	—
SANS Total, mean (sd)	—	24 (13)	41 (17)	**<0**.**001**						
General psychopathology (PANSS Total), mean (sd)	—	57 (16)	62 (14)	0.206	—	—	—	—	—	—
Positive symptoms (PANSS subset), mean (sd)		6 (3)	9 (6)	**<0**.**001**	—	—	—	—	—	—
CPZ equivalents, mean (sd) dose	—	338 (312)	953 (439)	**<0**.**001**	—	—	—	—	677 (506)	—
Clozapine plasma, mean (sd) µg/L	—	—	417 (240)	—	—	—	—	—	—	—
pH, mean (sd)	—	—	—	—	—	—	—	6.7 (0.3)	6.6 (0.3)	0.366
PMI, mean (sd) hours	—	—	—	—	—	—	—	26 (12)	282 (14)	0.705
Hemisphere, % (n) left	—	—	—	—	—	—	—	34 (13)	53 (20)	0.165

CPZ, chlorpromazine; PANNS, positive and negative syndrome scale; PMI, post-mortem interval; SANS, scale for the assessment of negative symptoms; TRS, treatment-resistant schizophrenia.

All procedures were conducted in accord with principles expressed in the Declaration of Helsinki and informed consent was obtained when required. Ethics approval for the postmortem brain studies was approved and conducted under the guidelines of the Human Research Ethics Committee at the University of New South Wales (HREC 07261). The recent-onset psychosis cohort recruitment and procedures were approved by the Melbourne Health Research Ethics Committee (MHREC ID 2012.066). The chronic schizophrenia cohort recruitment and procedures were approved by the Melbourne Health Research Ethics Committee (MHREC ID 2012.069).

### Clinical measures

The Structured Clinical Interview for DSM-IV Axis I Disorders^26^ or Mini International Neuropsychiatric Interview^27^ were used to confirm diagnosis. To measure negative symptom severity the Scale for the Assessment of Negative Symptoms (SANS)^28^ was used, and for general psychopathology and positive symptom severity the Expanded Brief Psychiatric Rating Scale (BPRS)^29^ or the Positive and Negative Syndrome Scale (PANSS)^30^ were used. BPRS scores were converted to PANSS scores as previously described^31^ to allow for consistent analysis of symptoms across cohorts. Current/last chlorpromazine equivalent dosage was calculated in all patients in all cohorts by following standard guidelines^32,33^, and clozapine plasma levels were measured from the treatment-resistant schizophrenia participants.

### UPS assessment

Levels of free mono-ubiquitin and ubiquitinated proteins as well as catalytic ubiquitination (i.e. E1 ubiquitin–activating enzymes, E2 conjugating enzymes, and E3 protein ligases) and proteasome (caspase-like, chymotrypsin-like and trypsin-like) activity were quantified in postmortem OFC and erythrocytes blind to diagnosis. All antibodies used in the current study are listed in Supplementary Table S1. Gels and blots were processed in parallel using the LI-COR Odyssey Infrared Imaging System Model 9120 and analyzed with image studio Lite Ver.4.0. Furthermore, to explore the potential effects of clozapine on ubiquitinated protein levels, mouse cortical neuronal cultures were prepared as described previously^34^. For details of the experimental procedures used for UPS assessment see supplementary material.

### Statistical analyses

Data were analysed using *R* 3.3.0 (R Foundation for Statistical Computing Vienna, Austria). To examine differences among the cohorts we used a linear regression approach. However, assumption testing revealed the presence of outliers and/or influential points for some of the models. Therefore, we fitted robust linear models and estimated the unstandardized beta (*b*) coefficient using an SMDM-estimator to control for the influence of outliers, heterogeneity^35,36^, and confounders (see supplementary materials for details on confounder analysis procedures).

We examined differences among the cohorts by first fitting a null model. This was either an intercept-only model or a covariate-only model if we were adjusting for confounding variables. We then fit a full model containing the cohort variable in addition to the terms in the null model. We compared the null and full models using the likelihood-ratio chi-squared test, with a significant test indicating that there were significant group differences between the cohorts.

We also conducted *post hoc* pairwise comparisons between the cohort groups, adjusted for multiple testing using the Benjamini-Hochberg (B-H) procedure, and computed Hedge’s g effect size based on the group means and standard errors adjusted for outliers and confounders, when the likelihood-ratio chi-squared test was significant. The *robustbase*^37^ package was used for the robust regression modelling, the *car*^38^ package was used for the likelihood-ratio chi-squared test, and the *lsmeans*^39^ package to calculate the adjusted group means, standard errors, and post hoc pairwise comparisons. To estimate clinical applicability, we also calculated the area under the receiver-operator curve (AUC), which is also known as the “common language effect size” or the “probability of superiority”^40^. This effect size gives the probability that a person picked at random from the comparison group will have a higher score than a person picked at random from the reference group^41^.

## Results

### Ubiquitinated protein levels are elevated in brain and erythrocytes in schizophrenia

**Figure 1:**
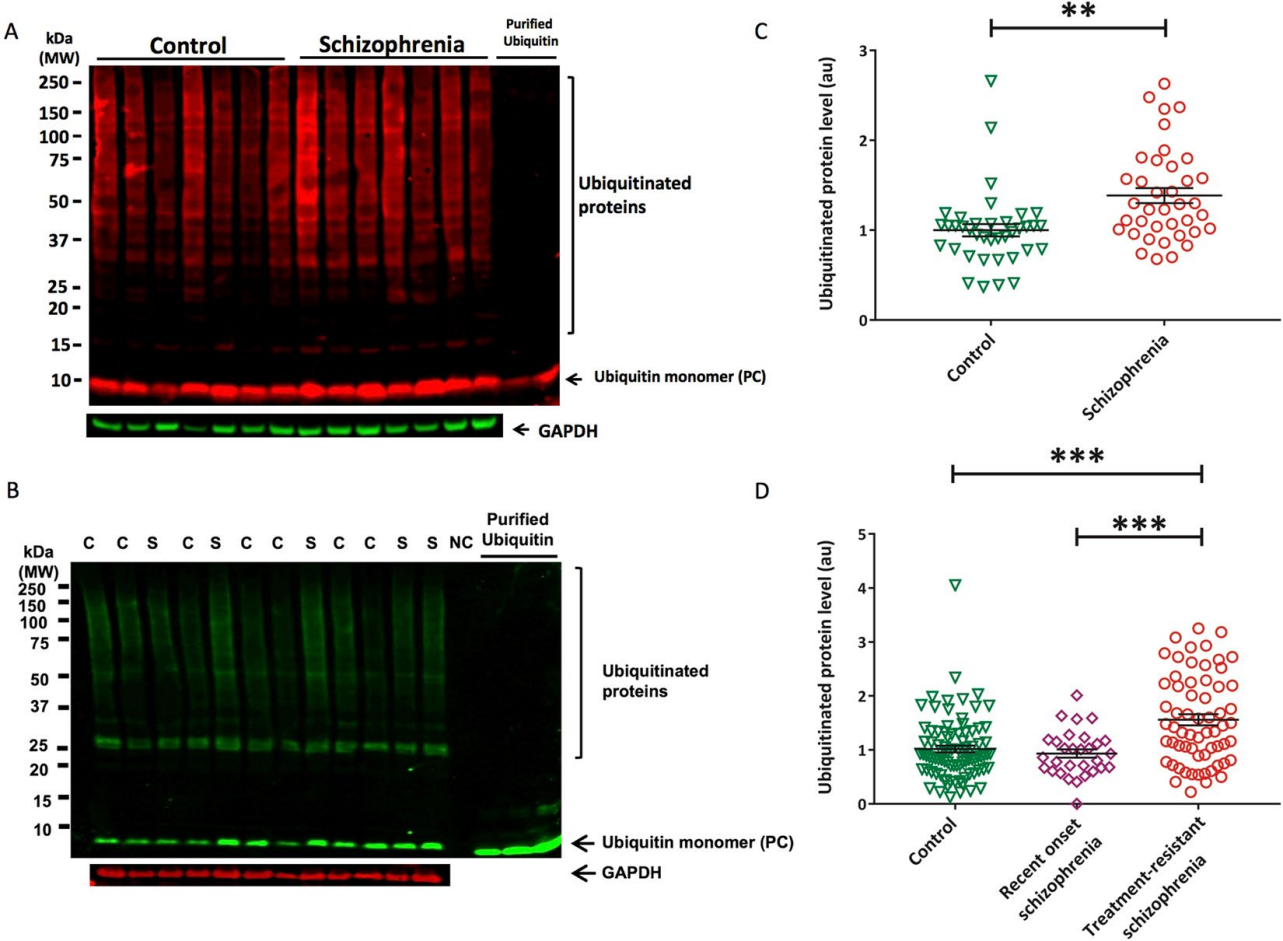
Elevated ubiquitinated proteins in erythrocytes and orbitofrontal cortex among those with schizophrenia. Example Western blots showing the quantified bands from 15–250 kD, indicating ubiquitinated proteins in orbitofrontal cortex (**A**) and erythrocytes (**B**). S, schizophrenia; C, control. All samples were normalized to the internal control (purified ubiquitin) to account for gel-to-gel variability. Ubiquitinated protein levels normalized to GAPDH in orbitofrontal cortex among those with schizophrenia (mean = 1.31, se = 0.08) and controls (mean = 0.94, se = 0.08) (**C**) as well as erythrocytes from those with treatment-resistant schizophrenia (mean = 1.48, standard error [se] = 0.09), recent onset schizophrenia (mean = 0.93, se = 0.12), and healthy controls (mean = 0.95, se = 0.07) (**D**). **p < 0.01, ***p < 0.001, NC = negative control, PC = positive control. Western blot images shown are cropped to show the proteins of interest. The western blots were derived under the same experimental conditions; the original full-length western blot images are shown in Supplementary Fig. S4.

In postmortem OFC we detected ubiquitinated protein levels that were 25.5% higher in those with schizophrenia compared to controls (p_B-H_ = <0.001, Hedge’s g = 0.91, AUC = 74.2%) (Fig. 1A,C, Supplementary Table S2). Likewise, erythrocytes from individuals with treatment-resistant schizophrenia had 33.1% and 34.5% higher levels of ubiquitinated proteins compared to those from individuals with recent onset schizophrenia (p_B-H_ < 0.001, g = 0.56, AUC = 65.5%) and healthy controls (p_B-H_ < 0.001, g = 0.72, AUC = 69.4%) (Fig. 1B,D, Supplementary Table S3). However, total protein by OFC tissue weight did not differ between the schizophrenia and control groups (p = 0.336, g = 0.24) (Supplementary Fig. S1, Supplementary Table S4).

These findings could not be better explained by current/last daily chlorpromazine equivalent dose in the recent-onset (p = 0.616), treatment-resistant (p = 0.908), or postmortem (p = 0.723) schizophrenia cohorts. Furthermore, clozapine plasma levels were not associated with ubiquitinated protein levels within the treatment-resistant schizophrenia cohort (p = 0.977) nor did we detect an effect of clozapine on ubiquitinated protein levels in mouse primary cortical neurons (1day: p = 0.362; 7 days: p = 0.127, Fig. S2). We also did not detect sex, age of illness onset, duration of illness, smoking status, or cannabis use effects on erythrocyte or OFC ubiquitinated protein levels nor was there an association between OFC ubiquitinated protein levels and postmortem interval or brain hemisphere. Associations were noted between OFC ubiquitinated protein levels and age (*b* = 0.008, SE = 0.001, p = 0.015) and pH (*b* = 0.008, SE = 0.001, p = 0.015), although inclusion of these factors in our linear regression models did not change our results. Within the treatment-resistant cohort, we also found that increased ubiquitinated protein levels were associated with an increase in general psychopathology (PANSS total: *b* = 0.018, SE = 0.007, p = 0.017, p_B-H_ = 0.10) and negative symptom severity (SANS total: *b* = 0.013, SE = 0.006, p = 0.026, p_B-H_ = 0.10).

### Endogenous ubiquitination activity is lower in erythrocytes but not brain in schizophrenia

**Figure 2:**
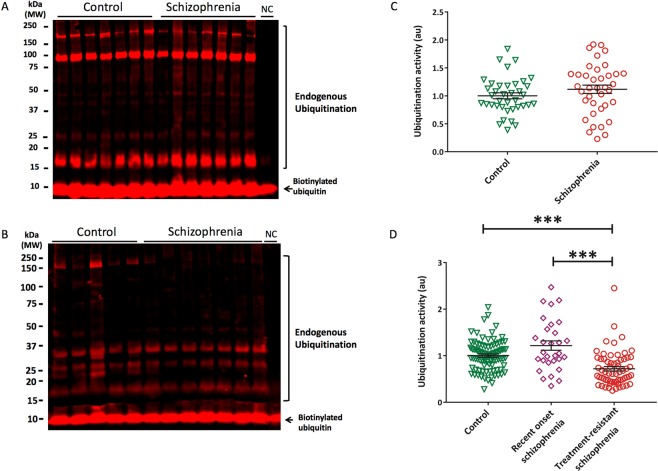
Decreased endogenous ubiquitination activity in erythrocytes but not orbitofrontal cortex among those with schizophrenia. Example Western blots showing the quantified bands from 15–250 kD, indicating endogenous ubiquitination activity in orbitofrontal cortex (**A**) and erythrocytes (**B**). All samples were normalized to the internal control (biotinylated ubiquitin) to account for gel-to-gel variability. Ubiquitination activity in orbitofrontal cortex among those with schizophrenia (mean = 1.13, se = 0.07) and controls (mean = 0.99, se = 0.07) (**C**) as well as among those with treatment-resistant schizophrenia (mean = 0.68, standard error [se] = 0.05), recent onset schizophrenia (mean = 1.14, se = 0.08), and healthy controls (mean = 0.99, se = 0.04) (**D**). *p < 0.01, NC = negative control, PC = positive control. Western blots images shown are cropped to show the proteins of interest. The western blots were derived under the same experimental conditions; the original full-length western blot images are shown in Supplementary Fig. S5.

To gauge the general status of the UPS machinery in red cells and brain tissues in schizophrenia, we employed a global polyubiquitination assay (termed “ubiquitination activity”) where the production of polyubiquitin chains from the net effects of the endogenous UPS machinery is assessed by incubating labeled mono-ubiquitin substrate with tissue lysates (as assay we term “ubiquitination ativity”). This activity was decreased in erythrocytes from individuals with treatment-resistant schizophrenia compared to healthy controls (p_B-H_ =< 0.001, g = 0.56, AUC = 65.5%) but was not changed in individuals with recent-onset schizophrenia (Fig. 2B,D). Ubiquitination ativity was also significantly lower in erythrocytes from the cohort with treatment-resistant schizophrenia compared to recent-onset schizophrenia (p_B-H_ =< 0.001, g = 0.62, AUC = 66.9%; Fig. 2B,D). There was no difference in ubiquitination ativity within the postmortem brain cohorts (p_B-H_ = 0.368, g = 0.28) (Fig. 2A,C).

We neither detected effects of sex, age of illness onset, duration of illness, smoking status, or cannabis use on erythrocyte or OFC ubiquitination activities, nor were there associations with chlorpromazine equivalent dose, postmortem interval, pH, or brain hemisphere in OFC tissue. Erythrocyte ubiquitination activity was positively correlated with age (*b* = 0.02, SE = 0.008, p = 0.020) and chlorpromazine equivalent dose (*b* = 0.02, SE = 0.008, p = 0.020) but adjustment for these factors in our regression model did not affect our main findings. Finally, ubiquitination activity was not associated with ubiquitinated protein levels in erythrocyte (*b* = 0.04, SE = 0.04, p = 0.292) or OFC (*b* = −0.15, SE = 0.10, p = 0.130).

### Brain and erythrocyte levels of free ubiquitin and proteasome activity do not differ in schizophrenia

Levels of free mono-ubiquitin and proteasome activity (chymotrypsin-like, trypsin-like and caspase-like) did not differ between healthy controls and those with schizophrenia in either the erythrocytes or OFC tissue (Supplementary Tables S2 and S3 and Supplementary Fig. S3). No effects of sex, age of illness onset, duration of illness, smoking status, cannabis use, or medication were found on erythrocyte or OFC ubiquitination activity, neither were there effects of postmortem interval or pH on OFC ubiquitination activity. However, increases in age were significantly associated with lower erythrocyte proteasome caspase-like (*b* = −0.03, SE = 0.003, p < 0.001), lower chymotrypsin-like (*b* = −0.003, SE = 0.001, p < 0.001), and higher trypsin-like (*b* = 0.01, *SE* = 0.001, *p* < 0.001) activities. In addition, OFC caspase-like (+37%, p = 0.012, g = 0.55) and chymotrypsin-like (+45%, p = 0.018, g = 0.59) activities were greater in the left compared to the right hemisphere among controls. Neither free ubiquitin levels nor proteasome activity were correlated with symptom severity.

## Discussion

**Figure 3:**
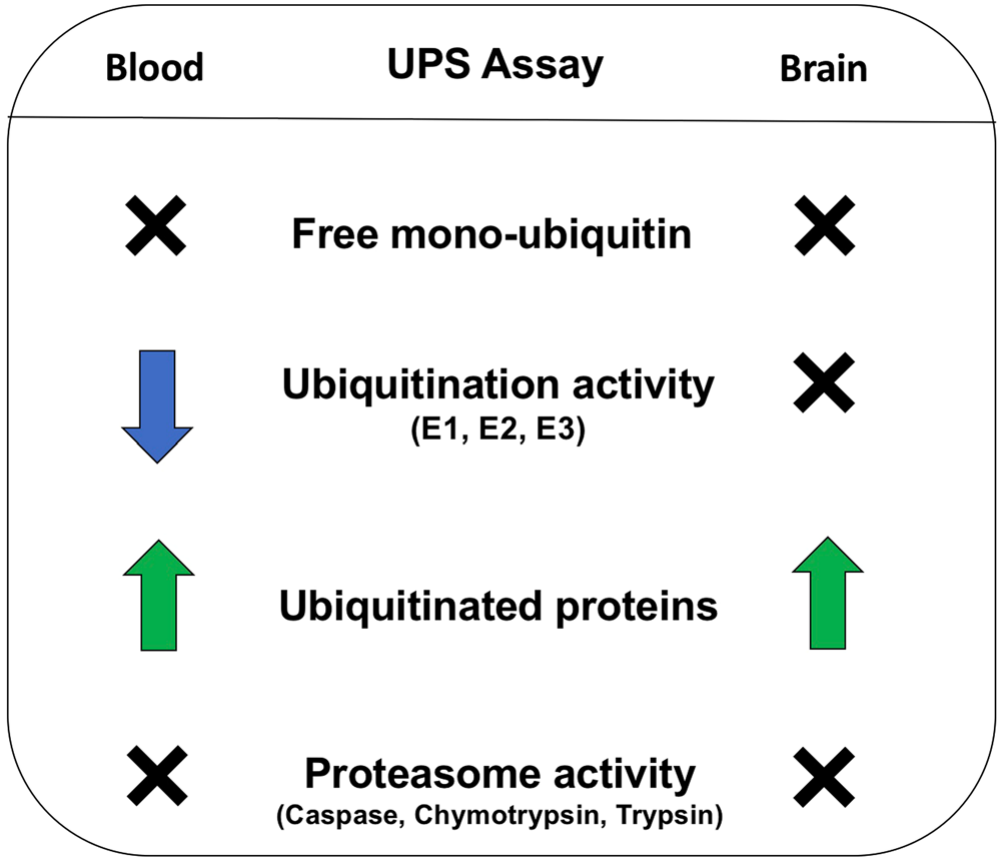
Summary of findings in erythrocytes and orbitofrontal cortex among those with schizophrenia relative to controls. X = no difference between schizophrenia and controls, down arrow = decreased in schizophrenia compared to controls, up arrow = increased in schizophrenia compared to controls.

We found significantly elevated levels of ubiquitinated proteins in the OFC and erythrocytes from individuals with treatment-resistant schizophrenia compared to controls, but a similar elevation was not detected in erythrocytes among those with recent-onset schizophrenia (Fig. 3). Our findings suggest that elevated ubiquitinated proteins are found later in the disease course, which might be consistent with increased protein denaturation as a consequence of chronic illness or of prolonged antipsychotic treatment. However, we failed to find evidence for an association of elevated ubiquitination with duration of illness, aligning with previous results^16^, and we did not detect any indication of medication effects within any of our cohorts or *in vitro* experiments. We then examined the effects of age, as aging is associated with a decrease in proteasome function, which in turn can increase ubiquitinated protein levels^42^. We found a positive correlation between age and ubiquitinated proteins in the OFC tissue but age alone does not account for our diagnostic increase in ubiquitination as our postmortem cases and controls were tightly matched on age. We did find the expected negative correlation between age and proteasome activity in erythrocytes, but did not detect a diagnostic difference in proteasome activity in either erythrocytes or OFC in schizophrenia compared to controls. Thus, while replicating the expected changes in the ubiquitination and proteasome systems with age, neither age nor proteasome dysfunction appear to be likely explanations for the observed elevation in ubiquitinated proteins in those with treatment-resistant schizophrenia.

Our results are at variance with previous postmortem results from the superior temporal gyrus of patients with schizophrenia who were ≈20 years older than our cohort, which described decreased protein levels of 19 S and 11 S proteasome regulatory particles in schizophrenia^17^. That report also found a decrease in both ubiquitinated proteins and ubiquitination enzymes in the superior temporal gyrus, which they attributed to a compensatory response to the proteasome’s diminished capacity for degrading ubiquitinated proteins^16^. Our findings provide only partial support for this explanation in that we found a decrease in ubiquitination activity in erythrocytes of those with schizophrenia but this was not recapitulated in the OFC. One potential explanation for the discordance is that the OFC is affected differently to superior temporal cortex. An alternative explanation is that in erythrocytes the only mechanism for protein degradation is via the UPS^18^, whereas in the OFC and other brain tissue protein degradation can also occur via the autophagosome-lysosomal system.

An elevation in ubiquitinated proteins in the presence of a normally functioning proteasome could also be attributed to abnormal deubiquitination^43,44^. Although we did not measure deubiquitination activity in the current study, three previous gene expression studies have implicated down-regulated expression of deubiquitinating enzymes in postmortem hippocampal neurons^7^ and prefrontal cortex tissue^9,10^ from those with schizophrenia. The deubiquitinating enzyme, *UCHL1*, was reported as down-regulated across these studies and in a phencyclidine rat model of schizophrenia^45,46^. However, brain lysates from Gracile axonal dystrophy mice, which lack *UCHL1*, do not show an accumulation of ubiquitinated proteins^47^, indicating a limited role, if any, in the elevation of ubiquitinated proteins in the current study. Nevertheless, the majority of known deubiquitinating enzymes are poorly characterized^48^ and, as such, further investigations of these enzymes, particularly those with tentative links to schizophrenia such as, *USP2*^13^, *USP9*^10^ and *USP14*^9^, are warranted.

Collectively, our results and those reported in the current literature do not point to a clear mechanism by which ubiquitinated proteins are increased in schizophrenia. However, we could not readily attribute this to abnormal proteasome function or an increase in ubiquitination activity. A decrease in deubiquitination activity remains unlikely given that free mono-ubiquitin levels were similar across all cohorts. Alternatively, the mechanism could be external to the UPS, such as oxidative stress. The UPS is involved in the degradation of damaged proteins generated by oxidative stress, which leads to increased levels of ubiquitinated proteins^49^. Oxidative stress is increased in schizophrenia^50–54^ and thought to be one of the mediators of neuroprogression, grey matter loss, and subsequent cognitive and functional impairment in the disorder^55^. Altered protein trafficking and turnover associated with oxidation-induced ubiquitination abnormalities in brain tissue may, in turn, contribute to neurotoxicity and functional impairment.

In addition to no clear mechanism, our results are unable to determine the likely outcomes of elevated ubiquitination in schizophrenia due to our global measurement of ubiquitination. In fact, previous work has shown that ubiquitination of a protein can result in an array of cellular effects (e.g. proteasomal degradation, modulation of signaling pathways, modification of protein function) depending on the length (i.e. mono or poly) and residue site (e.g. K48, K63) of the ubiquitin chains^56^. Thus, the elevation in ubiquitinated proteins we observed in the current study could reflect any one or more of these cellular outcomes. However, previous postmortem findings by Rubio *et al*.^16^ showed elevated levels of K63-linked and decreased levels of K48-linked poly-ubiquitinated proteins in schizophrenia relative to controls, implicating signaling pathway modulation, NF-kB activation, and/or DNA repair, rather than proteasomal degradation, may be perturbed in schizophrenia^56^. Nevertheless, additional work to discern whether the elevated levels of ubiquitinated proteins in schizophrenia are primarily mono- or poly-ubiquitinated as well as the distribution of the various ubiquitin chain types is  necessary for firm conclusions to be drawn.

In summary, we found elevated ubiquitinated proteins in erythrocytes and postmortem OFC tissue from individuals with schizophrenia. The explanation for and effects of this elevation are not clear. However, our results taken together with previous findings suggest that ubiquitination may be abnormal in both the brain and blood of those with schizophrenia, perhaps more clearly in the later stages or in specific subgroups (such as treatment-resistant) of the illness. Therefore, we propose that the accumulation of ubiquitinated proteins may correspond to a pathological marker that characterizes the latest stages of schizophrenia and may be linked to the accumulation of other proteins, such as DISC1, that has been found accumulated in the brain insoluble fractions of individuals with schizophrenia^57^. Thus, the observed ubiquitinopathy in this report may represent a convergence between protein misassembly and aggregation that has been suggested to explain the causes of chronic mental illness^57^. Follow-up studies to further refine the mechanism by which this abnormality in ubiquitination arises, or whether the erythrocyte abnormality may serve as a biomarker, are warranted.

## Supplementary information

Supplementary Materials
