# Supplementary material for: Elevated ubiquitinated proteins in brain and blood of individuals with schizophrenia
Source: Sci Rep. 2019 Feb 19;9:2307. doi: 10.1038/s41598-019-38490-1 (PMC6381171; doi:10.1038/s41598-019-38490-1)
Supplement: Supplementary file 1 — Supplementary Materials [file 41598_2019_38490_MOESM1_ESM.pdf]

SUPPLEMENTARY MATERIAL

Elevated ubiquitinated proteins in brain and blood of individuals with schizophrenia

Chad A. Bousman, PhD[1,2,11,12,13], Sandra Luza, PhD[1,3], Serafino G. Mancuso, PhD[1], Dali Kang, BSc[1,4], Carlos M. Opazo, PhD[3], Md Shaki Mostaid, MSc[1,2], Vanessa Cropley, PhD[1], Patrick McGorry, MD[5,7], Cynthia Shannon-Weickert, PhD[1,6,8], Christos Pantelis, MD[1,2,3,7,9], Ashley I. Bush, MBBS PhD[2,3], Ian P. Everall, MBChB PhD[1,2,3,9,10]

[1] Melbourne Neuropsychiatry Centre, Department of Psychiatry, The University of Melbourne & Melbourne Health; Parkville, VIC, Australia
[2] The Cooperative Research Centre (CRC) for Mental Health, Victoria, Australia
[3] Florey Institute of Neuroscience and Mental Health, The University of Melbourne, Parkville, VIC, Australia
[4] Guangdong Food and Drug Vocational College, Guangzhou, China
[5] Orygen, The National Centre of Excellence in Youth Mental Health, VIC, Australia
[6] Schizophrenia Research Laboratory, Neuroscience Research Australia, Baker Street, Sydney, Australia
[7] NorthWestern Mental Health, Melbourne, Victoria, Australia
[8] School of Psychiatry, Faculty of Medicine, University of New South Wales, Sydney, Australia
[9] Centre for Neural Engineering, The University of Melbourne, Carlton, VIC, Australia
[10] Institute of Psychiatry, Psychology and Neuroscience, King's College London
[11] Departments of Medical Genetics, Psychiatry, Physiology & Pharmacology, University of Calgary, Calgary, AB, Canada
[12] Alberta Children's Hospital Research Institute, University of Calgary, Calgary, AB, Canada
[13] Hotchkiss Brain Institute, Cumming School of Medicine, University of Calgary, Calgary, AB, Canada

Table of Contents

Supplemental Methods 3

Human postmortem ascertainment and exclusions 3

Recent onset psychosis participant recruitment 3

Treatment-resistant schizophrenia recruitment 4

Tissue collection and processing 5

UPS Assessment 5

Statistical Analysis 8

Supplementary Tables 10

Table S1. Antibodies used for Western blot analysis 10

Table S2. Comparison of UPS markers in post-mortem orbitofrontal cortex by diagnosis 11

Table S3. Comparison of UPS markers in controls, recent onset and treatment-resistant schizophrenia 12

Table S4. Comparison of total protein concentration in post-mortem orbitofrontal cortex by diagnosis 13

Supplementary Figures 14

Figure S1. Total proteins by wet weight in the orbitofrontal cortex among controls and those with schizophrenia. 14

Figure S2. Effect of Clozapine on the levels of ubiquitinated proteins in mouse primary cortical neurons 15

Figure S3. Free mono-ubiquitin levels in erythrocytes and orbitofrontal cortex among those with schizophrenia and controls 16

References 17

Human postmortem ascertainment and exclusions

Cases with an unclear psychiatric diagnosis, evidence of cocaine or phencyclidine (PCP) abuse by history and/or toxicology, cerebrovascular disease, autolysis, subdural hematoma, neuritic pathology or other pathological features were excluded from the cohort. Unaffected controls were screened by telephone interviews of family members and/or police records for a history of medical and/or psychiatric problems, including alcohol abuse and illicit drug use. Any positive history of a psychiatric problem or excessive alcohol or drug use led to the exclusion of that participant from the normal control group.

Orbitofrontal cortex (OFC) tissue from people with schizophrenia or schizoaffective disorder (n=38) and healthy controls (n=38), matched for age, brain pH and postmortem interval (PMI), was obtained from the New South Wales Brain Tissue Resource Centre (Sydney, Australia). In RNAse-free conditions, blocks for tissue sections (14 μm) and for pulverisation/homogenisation were dissected from fresh frozen coronal slabs of OFC which were identified as being on the ventral surface below the inferior frontal gyrus, and anterior to the appearance of the lateral ventricle. All blocks for sectioning contained gyrus rectus (Brodmann area 11) on the medial side. For homogenisation, approximately 0.5 g of predominantly grey matter tissue was excised from the most lateral end of the larger OFC block corresponding to areas 47/12 (Öngür et al, 2003) on a dry ice platform using a dental drill (Cat# UP500-UG33, Brasseler, USA) at moderate speed (up to 40,000 rpm) to minimize heat generation during the dissection. Tissue from each case was then pulverised over a frozen tray placed in dry ice, weighed while frozen, and stored at –80°C until protein was extracted.

Recent onset psychosis participant recruitment

Thirty recent onset patients and 31 unrelated healthy control participants were recruited from multiple clinical services and the community in Melbourne, Australia as part of an on-going neuroimaging study. Individuals were eligible to participate if they were aged between 18-50

years and recent onset individuals required a DSM-IV Axis I diagnosis of a first episode psychotic disorder. Exclusion criteria for both recent onset and controls included: 1) history of significant head injury, neurological disease, or seizures, 2) current pregnancy/breastfeeding, 3) impaired thyroid function, 4) diabetes, 5) Daily use of steroidal or nonsteroidal anti-inflammatory, immunosuppressive, corticoid or glucocorticoid drugs continuously for more than one week within the last month, 6) generalized inflammatory condition/disease, or 7) premorbid IQ less than 70 with a documented history of developmental delay or intellectual disability. In addition, recent onset individuals were excluded if psychotic symptoms were entirely explained by drug use and healthy controls were excluded if they had a personal or family history of psychiatric illness, had significant psychopathology according to the Structural Clinical Interview for DSM-IV Axis I Disorders (SCID)[1] or Comprehensive Assessment of At Risk Mental States (CAARMS)[2], or were currently taking psychotropic medications.

Treatment-resistant schizophrenia recruitment

Sixty-three participants with treatment-resistant schizophrenia (TRS) were recruited from inpatient and outpatient clinics located in Melbourne, Australia. Inclusion criteria included: 1) diagnosis of schizophrenia, 2) currently prescribed and taking clozapine and 3) aged between 18-65 years. Fifty-seven unrelated healthy controls matched for age and sex with similar socio-economic backgrounds were recruited from the general community. Controls with a first-degree family history of psychiatric illness, neurological disease, head injury, seizures, prior or current use of antipsychotic medication, impaired thyroid function and/or substance abuse/dependence were excluded from the study. All participants were administered the Mini International Neuropsychiatric Interview (MINI)[3] to confirm the diagnosis of schizophrenia and to rule out current or past psychiatric illness in healthy controls.

Tissue collection and processing

Postmortem OFC samples were homogenized 1/5 (w/v) in 50 mM Tris HCl pH 7.5 containing 1% (v/v) NP-40, 10mM dithiothreitol (DTT), NaCl 150 mM, 10 mM N-ethylmaleimide (NEM), protease inhibitors cocktail without EDTA (Roche, USA) and phosphatase inhibitors cocktail (Roche, USA). The homogenized samples were centrifuged at 10,000 g for 10 min at 4^0C. Then supernatants (NP40-soluble fraction) were collected and protein concentrations were determined using the BCA Protein Assay Kit (Pierce, USA) and supernatants were aliquoted and stored at -80°C until use.

For both the recent onset and treatment-resistant schizophrenia cohorts (including controls), whole blood was collected after overnight fasting. Samples were spun at 200 g, 20 °C for 10 minutes, with no brake. The platelet rich plasma was removed from the red blood cell fraction with a plastic transfer pipette the red blood cell fractions were washed by adding 0.9% normal saline to an end volume of approximately 14 mL. The red blood cells were then gently disperse by inverted the tube 10 times, and then centrifuge at 650 g for 10 minutes at 20 ^{o}C with brake. This procedure was repeated three times, removing the saline wash each time (the final wash spun at 1,500 g for 10 minutes with brake in order to pack the red blood cells more tightly and minimise the volume of saline left behind. The final saline wash was discarded and the cells are resuspended in an end volume of 6 mL PBS (pH 7.4), then aliquoted and stored in liquid nitrogen. Erythrocytes were lysed in NP-40 buffer described above minus 10mM DTT. The lysates were centrifuged and protein concentrations were determined in the NP-40 soluble fraction as described above and then stored at -80°C until use.

UPS Assessment

Levels of free mono-ubiquitin and ubiquitinated proteins. Erythrocyte NP40-soluble fractions (30 μg) of each sample were electrophoretically separated by 4-12% SDS-polyacrylamide gels for 1h at 120 V. A standard curve of ubiquitin (0.05-0.2 μg) purified from bovine erythrocytes (Sigma-Aldrich, USA) was included in all gels. The proteins were

transferred to a PVDF membrane for 1h at 20 V. The blots were blocked with 5% skim milk in Tris-buffered saline containing 0.1% Tween-20 (TBST), and were then incubated for 16 h with the primary antibodies diluted in TBST containing 3% BSA (polyclonal rabbit anti-*Ubiquitin* antibody (1: 500, DAKO, Denmark) and mouse monoclonal GADPH (1:10,000) at 4^0C. After four washings with TBST, the blots were incubated with conjugated secondary antibodies (1:10,000, Li-Cor, USA) in TBST, 0.1% SDS for 1h at 22^0C, protected from light during the incubation. After four washes, the blots were finally immersed in PBS and the infrared signal detected in a LiCor imager (Li-Cor, Lincoln, USA).

Densitometry was performed by a blinded operator. Two densitometry boundary boxes were manually placed around each band of expected molecular weight (~8.5 kDa and ~15 to ~250 kDa) to obtain integrated intensity values acquired in a Li-Cor imager using the Odyssey 4.0 analytical software. Unconjugated ubiquitin (~8.5 kDa) was identified and quantified as free ubiquitin. Bands between 15 to 250 kDa were considered as ubiquitinated proteins for the quantitation. The signal of the region of interest was transformed to ubiquitin levels (μg) using the ubiquitin standard curve that was included in each gel (see above). The levels of GAPDH were used as a loading control. However, the results and analysis were unaltered by this correction. The values of the samples were normalized to the average value of the control samples analyzed in each gel.

To explore the potential effects of clozapine on ubiquitinated protein levels, mouse cortical neuronal cultures, which express D2 receptors [4], were prepared as described previously [5] in accordance with animal ethics committee approval of the The Florey Institute of Neuroscience and Mental Health Research. Briefly, cortices were removed, dissected free of meninges and dissociated in 0.025% (w/v) trypsin. Dissociated cells were plated in poly-l-lysine coated sterile 6 well culture plates in minimal essential medium (MEM) supplemented with 10% fetal calf serum. Cultures were maintained at 37°C in 5% CO_2 for 2 h before the plating medium was replaced with Neurobasal growth medium containing B27 supplements (Life

Technologies Inc., USA). After 7 days in culture, cells were treated in duplicate with clozapine (Sigma-Aldrich, St. Louis, Missouri, USA) at a concentration of 1.2μM (control wells exposed to vehicle only) for 1 or 7 days in Neurobasal media supplemented with B27. Then, total levels of ubiquitinated proteins found in NP40-soluble fractions were detected by Western blot using the polyclonal rabbit anti-*Ubiquitin* antibody (1: 500, DAKO, Denmark). Levels of β-actin were used as internal loading control. 10 μg of protein were loaded in each lane.

Endogenous ubiquitination activity. Postmortem and erythrocyte NP40-soluble fractions (10 μg) were incubated for 1 h at 37^{0}C in the presence of a master mix (Tris- HCl 50 mM, DTT 1 mM, $MgCl_2$ 5 mM, ATP 5 mM and biotinylated-ubiquitin 2.5 μM (Enzo Life Sciences Inc., USA) to measure the total endogenous ubiquitination enzymatic activity, which is visualized as the conjugation of biotinylated-ubiquitin with the E1, E2, E3 enzymes and the substrates present in the biological sample. The reaction was stopped with non-reducing sample buffer (2x). The samples were heated for 5 mins at 90^{0}C and the proteins were separated on 4-12% SDS-polyacrylamide gels for 1h at 120 V. Then, the proteins were transferred to a PVDF membrane for 1h at 20 V. The blots were blocked with 1% BSA in Tris-buffered saline containing 0.1% Tween-20 (TBST), and were then incubated for 1h at 22^{0}C with Streptavidin- 680IR (Li-Cor, Lincoln, USA), protected from light during the incubation. Then, the blots were washed four times with TBST for 10 mins each. The blots were finally immersed in PBS and the infrared signal detected in a LiCor imager. The signal between ~15 to ~250 kDa was considered as ubiquitinated protein products of the ubiquitination activity for the quantitation. Thus, a densitometry boundary box was manually placed between ~15 to ~250 kDa in each lane to obtain integrated intensity values acquired in a LiCor imager using the Odyssey 4.0 analytical software. Data were expressed as arbitrary units and the values were normalized to the average signal of the control samples.

Proteasome activity. Samples (≈10 mg (wet weight) of post-mortem OFC brain, or 20 μl of erythrocytes) were homogenized in 10 mM HEPES (pH= 7.6) and centrifuged at 10,000 g for

10 min at 4°C. The supernatant was separated and the protein concentration determined as described above. Optimal concentrations of the samples were determined for each enzymatic assay, and the samples adjusted with 10 mM HEPES (pH= 7.6) to final working concentrations: chymotrypsin-like activity 0.2 µg/µl, trypsin-like activity 2 µg/µl, and caspase-like activity 0.4 µg/µl. Proteasome activities (chymotrypsin-like, trypsin-like and caspase-like) were assayed using the Proteasome-Glo kit (Promega, USA) in 384-well plates for luminescent assays. Twenty-five µl of each specific protease reagent was added to 25 µl of each sample. A blank (10 mM HEPES; pH=7.6) as well as a standard curve of purified 26S Proteasome (0.5, 1 and 10 g/L; Enzo Life Sciences Inc., USA) were included in each assay. Each enzymatic activity was measured after 20 minutes of incubation when the signal was in the dynamic range (Promega, USA): the luminescent signal of each well was detected and quantified using a FlexStation 3 reader (Molecular Devices, USA). Data are expressed as arbitrary units related to 26S standard curve activity.

Statistical Analysis

Prior to our main analysis, we assessed the following factors as potential confounders: chlorpromazine equivalent dose, clozapine plasma levels (treatment-resistant cohort only), age of illness onset, duration of illness, cannabis use (recent onset and treatment-resistant cohorts only), and smoking status. In the post-mortem cohort we also assessed OFC pH, post-mortem interval, and brain hemisphere as potential confounds.

We used a new forward stepwise-based procedure [6] that uses a step-by-step method to select the best combination of covariates and uses bootstrapped resampling methods to determine the number of covariates to include in the model. The model containing the cohort variable with the smallest deviance statistics was then used to examine cohort differences. The *FWDselect* package [7] was used for this model selection approach.

Furthermore, among cases in the clinical and post-mortem cohorts, a series of regressions were conducted to determine whether chlorpromazine equivalent levels, age of illness onset, or illness duration were related to any of the UPS markers as well as potential associations between clozapine plasma levels and UPS markers in the treatment-resistant cohort.

Table S1: Antibodies used for Western blot analysis

Antibody	Host	Dilution	Buffer	Company	Catalogue #	Location
Ubiquitin	Rabbit	1:500	TBST	Dako	Z 0458	Denmark
Ubiquitin P4D1	Mouse	1:500	TBST	Santa Cruz Biotechnology	Sc-8017	USA
GAPDH	Mouse	1: 10,000	TBST	Sigma-Aldrich	G8795	USA
GAPDH	Rabbit	1:20,000	TBST	Sigma-Aldrich	G9545	USA
Anti-mouse IRDye (680RD; 800CW)	Goat	1:10,000	TBST	Li-Cor, Lincoln	ab216776 ab216772	USA
Anti-rabbit IRDye (680Rd; 800CW)	Goat	1:10,000	TBST	Li-Cor, Lincoln	ab216777 ab216773	USA

Table S2. Comparison of UPS markers in post-mortem orbitofrontal cortex by diagnosis

	Control		Schizophrenia		Test		
**UPS marker**	***M***	***SE***	***M***	***SE***	***p***	***g***	**AUC**
Free Ub[a]	0.98	0.05	1.11	0.05	0.230	-0.42	61.7%
Ub enzymes[a,b,c,d]	1.05	0.08	1.16	0.07	0.368	-0.28	57.9%
Ub proteins[a]	0.99	0.06	1.33	0.06	**<0.001**	-0.91	74.2%
Caspase-like[b,c,e]	1.05	0.09	1.13	0.07	0.439	-0.19	55.4%
Trypsin-like	10.79	0.64	9.97	0.55	0.402	0.23	56.7%
Chymotrypsin-like[a,c,d,e]	0.36	0.03	0.41	0.02	0.311	-0.36	60.1%

Note. All means and standard errors are adjusted for outliers.
p-values adjusted for multiple testing using FDR correction.
g = Hedge's *g* and AUC = area under the receiver-operator curve.
[a] Adjusted for OFC pH.
[b] Adjusted for hemisphere.
[c] Adjusted for sex.
[d] Adjusted for age at death.
[e] Adjusted for lifetime smoking status.

Table S3. Comparison of UPS markers in controls, recent onset and treatment-resistant schizophrenia

	**1. Control**		**2. Recent Onset**		**3. TRS**		**Omnibus**	**1 vs 2**			**1 vs 3**			**2 vs 3**		
**UPS marker**	***M***	***SE***	***M***	***SE***	***M***	***SE***	***p***	***p***	***g***	**AUC**	***p***	***g***	**AUC**	***p***	***g***	**AUC**
Free Ub[a,b]	0.96	0.09	0.58	0.16	0.89	0.11	0.247	—	—		—	—		—	—	
Ub enzyme activity	0.98	0.04	1.11	0.07	0.76	0.04	**<0.001**	0.119	-0.23	56.4%	**<0.001**	0.56	65.5%	**<0.001**	0.62	66.9%
Ub proteins[c]	0.94	0.07	0.90	0.12	1.43	0.08	**<0.001**	0.790	0.04	51.1%	**<0.001**	-0.72	69.4%	**<0.001**	-0.56	65.5%
Caspase-like[c,d]	1.09	0.03	1.01	0.05	1.08	0.03	0.491	—	—		—	—		—	—	
Trypsin-like[a,d,e]	0.99	0.02	0.98	0.03	0.97	0.02	0.715	—	—		—	—		—	—	
Chymotrypsin-like[d]	0.323	0.003	0.329	0.006	0.327	0.004	0.497	—	—		—	—		—	—	

Note. All means and standard errors are adjusted for outliers.
p-values adjusted for multiple testing using FDR correction.
g = Hedge's *g* and AUC = area under the receiver-operator curve.
[a] Adjusted for current smoking status.
[b] Adjusted for current cannabis use.
[c] Adjusted for sex.
[d] Adjusted for assay batch.
[e] Adjusted for age.

Table S4. Comparison of total protein concentration in post-mortem orbitofrontal cortex by diagnosis

	**Control**		**Sz**		**Test**		
	M	***SE***	***M***	***SE***	***p***	***g***	**AUC**
Total Proteins	20.27	0.83	21.31	0.65	0.336	-0.24	56.9%
Ub proteins/Total Proteins	0.045	0.004	0.061	0.003	**0.012**	-0.72	69.7%
Total Proteins/Wet Weight (mg/mg)	0.122	0.005	0.128	0.004	0.336	-0.24	56.9%

Note. All means and standard errors have been adjusted for outliers and heterogeneity.
All means and standard errors have also been adjusted for sex, age at death, lifetime smoking status, OFC pH, hemisphere, and PMI.
p-values adjusted for multiple testing using FDR correction.
g = Hedge's *g* and AUC = area under the receiver-operator curve.

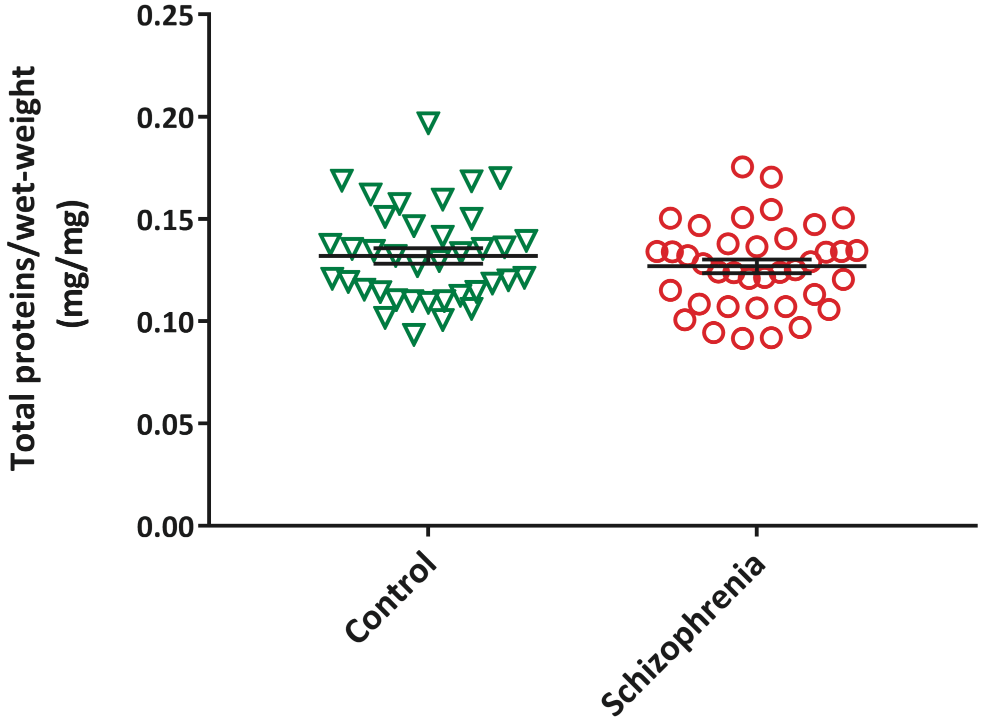

Figure S1. Total proteins by wet weight in the orbitofrontal cortex among controls and those with schizophrenia.

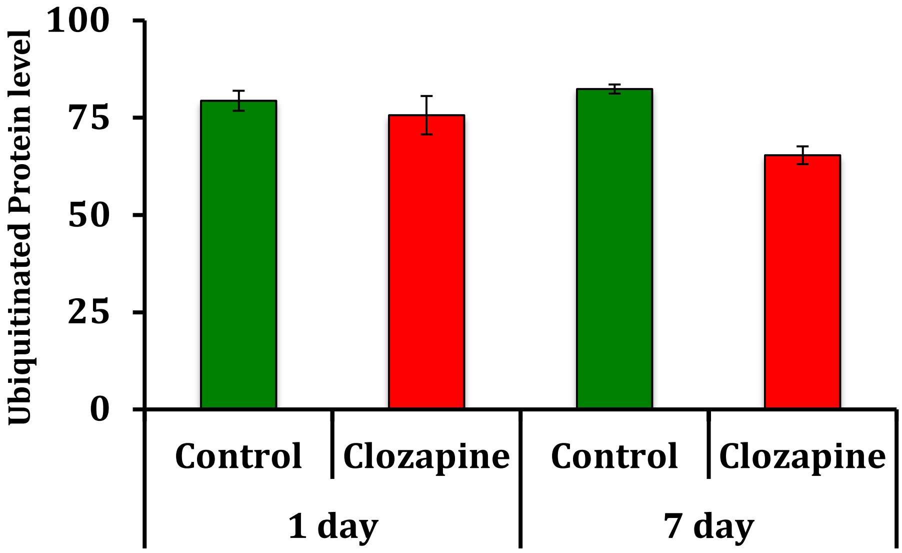

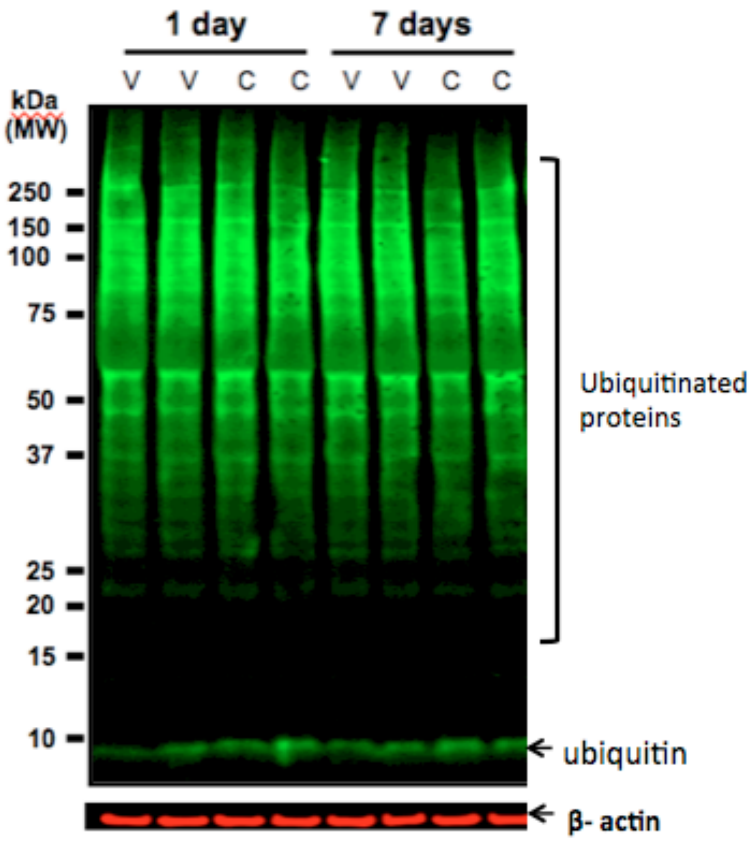

Figure S2. **Effect of Clozapine on the levels of ubiquitinated proteins in mouse primary cortical neurons.** Cells were treated in duplicate with clozapine (1.2 μM) or vehicle (controls) for 1 or 7 days in Neurobasal media supplemented with B27. Then, total levels of ubiquitinated proteins (Ubp; green) were detected by Western blot using an anti-ubiquitin polyclonal antibody. Levels of β-actin (red) were used as internal loading control. 10 μg of protein were loaded in each lane. V=vehicle, C=Clozapine.

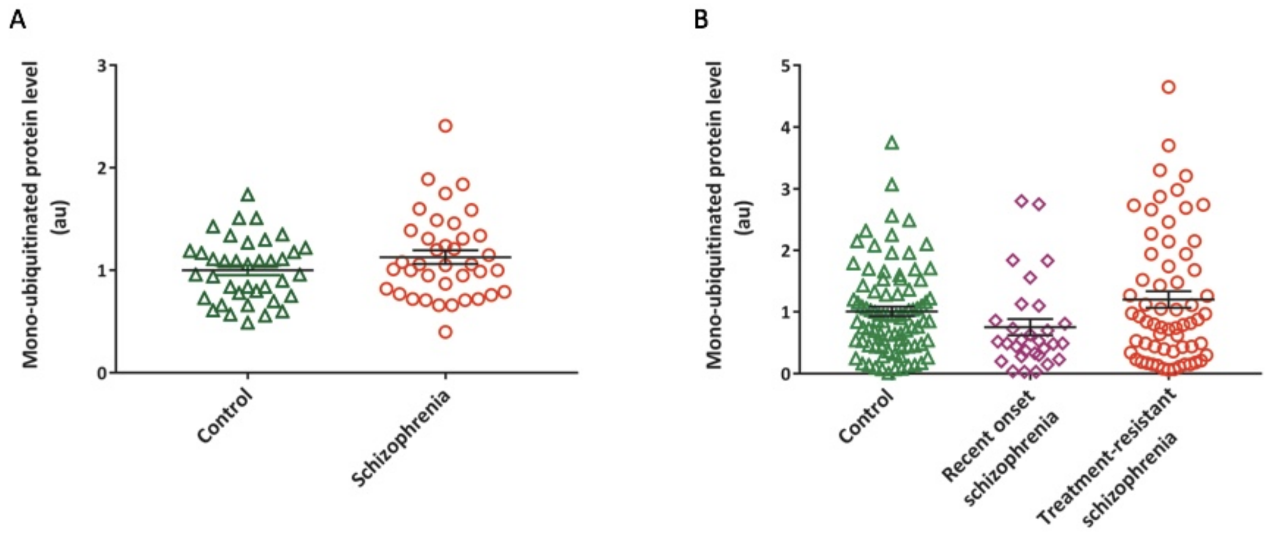

Figure S3. Free mono-ubiquitin levels in orbitofrontal cortex (A) and erythrocytes (B).

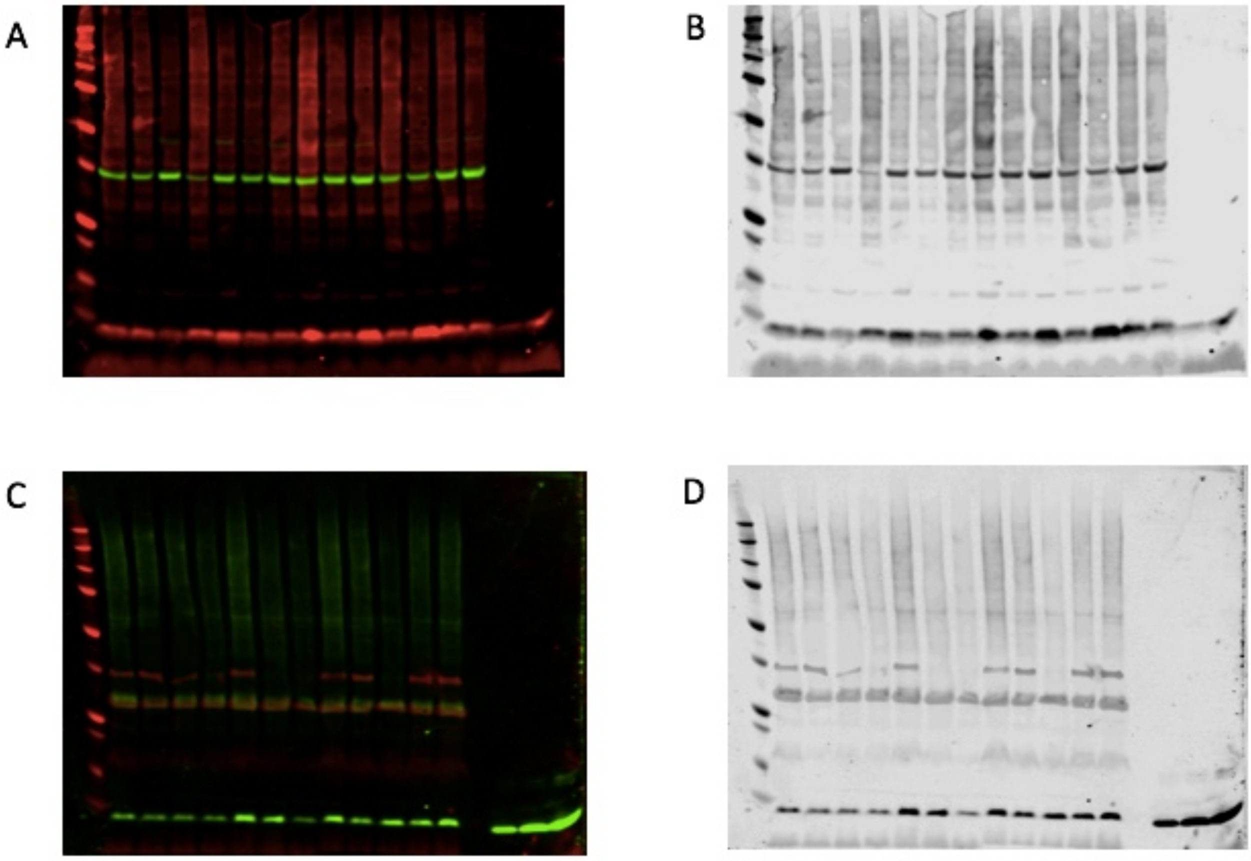

Figure S4. Full-length blots for ubiquitinated proteins in erythrocytes (A, B) and orbitofrontal cortex (C, D) among those with schizophrenia.

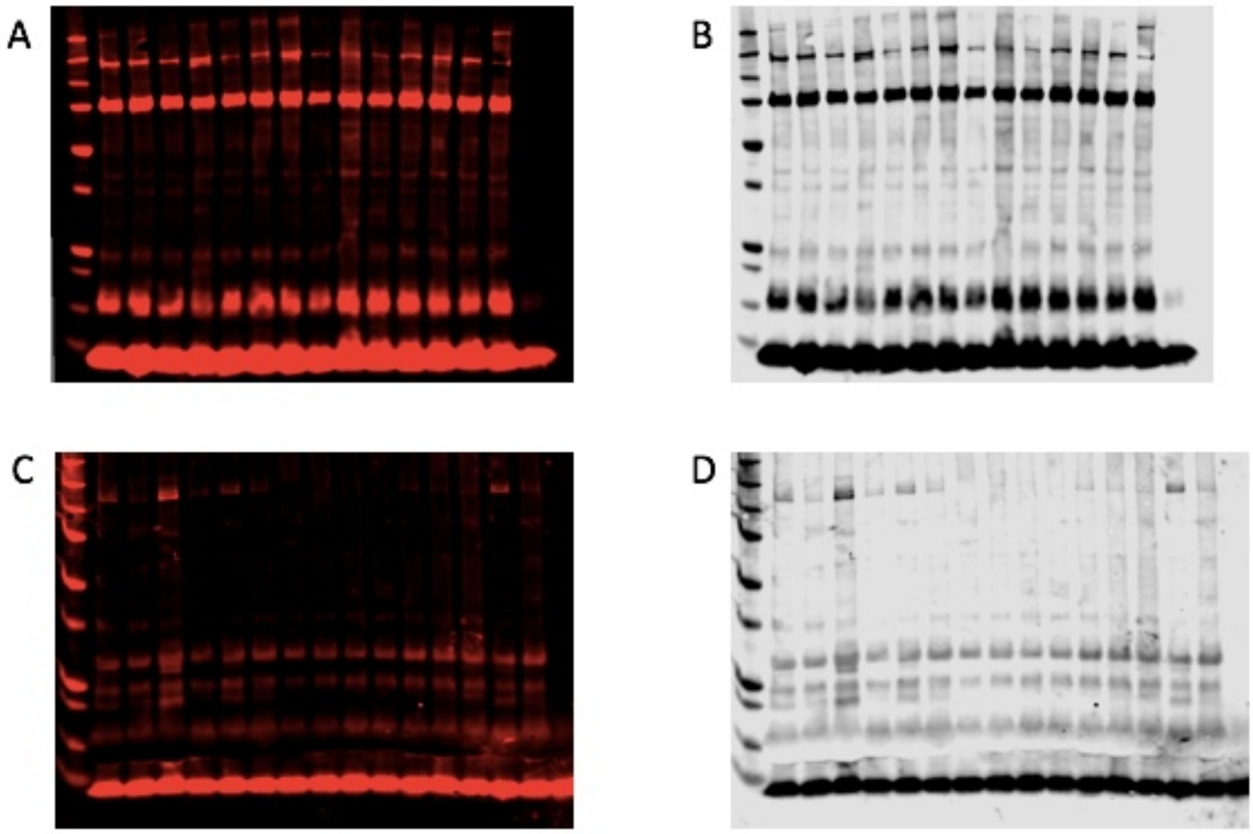

Figure S5. Full-length blots for endogenous ubiquitination activity in erythrocytes (A, B) but not orbitofrontal cortex (C,D) among those with schizophrenia.

References

1 First, M. B., Gibbon, M., Spitzer, R. L. & Williams, J. B. *Structured Clinical Interview for DSM-IV Axis I Disorders.*, (American Psychiatric Publishing, Inc., 1997).

2 Yung, A. R. *et al.* Mapping the onset of psychosis: the Comprehensive Assessment of At-Risk Mental States. *Aust N Z J Psychiatry* **39**, 964-971, doi:10.1111/j.1440-1614.2005.01714.x (2005).

3 Sheehan, D. V. *et al.* The Mini-International Neuropsychiatric Interview (M.I.N.I.): the development and validation of a structured diagnostic psychiatric interview for DSM-IV and ICD-10. *The Journal of clinical psychiatry* **59 Suppl 20**, 22-33;quiz 34-57 (1998).

4 Araki, K. Y., Sims, J. R. & Bhide, P. G. Dopamine receptor mRNA and protein expression in the mouse corpus striatum and cerebral cortex during pre- and postnatal development. *Brain Res* **1156**, 31-45, doi:10.1016/j.brainres.2007.04.043 (2007).

5 Opazo, C. *et al.* Metalloenzyme-like activity of Alzheimer's disease beta-amyloid. Cu-dependent catalytic conversion of dopamine, cholesterol, and biological reducing agents to neurotoxic H(2)O(2). *J Biol Chem* **277**, 40302-40308, doi:10.1074/jbc.M206428200 (2002).

6 Sestelo, M., Villanueva, N. M., Meira-Machado, L. & Roca-Pardiñas, J. FWDselect: An R Package for Variable Selection in Regression Models. *R Journal* **8** (2016).

7 FWDselect: Selecting Variables in Regression Models v. R package version 2.1.0 (2015).
